# An Electronic Health Intervention for Latina Women Undergoing Breast Cancer Treatment (My Guide for Breast Cancer Treatment): Protocol for a Randomized Controlled Trial

**DOI:** 10.2196/14339

**Published:** 2019-12-13

**Authors:** Betina Yanez, Sharon H Baik, Laura B Oswald, Diana Buitrago, Joanna Buscemi, Francisco Iacobelli, Alejandra Perez-Tamayo, Precilla Fajardo, Gabriela Serrano, Judith Guitelman, Frank J Penedo

**Affiliations:** 1 Northwestern University Feinberg School of Medicine Chicago, IL United States; 2 DePaul University Chicago, IL United States; 3 Northeastern Illinois University Chicago, IL United States; 4 University of Illinois at Chicago Chicago, IL United States; 5 ALAS-WINGS Chicago, IL United States; 6 University of Miami Miami, FL United States

**Keywords:** breast cancer, Latina, health-related quality of life, eHealth, randomized controlled trial

## Abstract

**Background:**

Among Latinas and Hispanics (henceforth referred to as Latinas), breast cancer is the most commonly diagnosed cancer and the leading cause of cancer-related deaths. However, few interventions have been developed to meet the needs of Latina women undergoing active treatment for breast cancer.

**Objective:**

This paper aims to describe the procedures and methods of *My Guide for Breast Cancer Treatment* and the plans for conducting a multisite randomized controlled trial to investigate the feasibility and preliminary efficacy of this smartphone-based app for Latina women in active treatment for breast cancer.

**Methods:**

Study participants will be randomized to the *My Guide for Breast Cancer Treatment* intervention or the enhanced usual care control condition for 12 weeks. Participants will have access to innovative features such as gamification via virtual awards to reinforce usage and an adaptive section that presents targeted material based on their self-reported concerns and needs. Using a stepped-care approach, intervention participants will also receive telecoaching to enhance their adherence to the app. Study outcomes and intervention targets will be measured at study enrollment (before randomization), 6 and 12 weeks after initial app use. General and disease-specific health-related quality of life (HRQoL) and symptom burden are the study’s primary outcomes, whereas anxiety, depression, fear of cancer recurrence, physical activity, and dietary intake are secondary outcomes.

**Results:**

Recruitment began in August 2019 and is expected to be completed by August 2020. We expect to submit study results for publication by fall 2020.

**Conclusions:**

*My Guide for Breast Cancer Treatment* has the potential to improve HRQoL and reduce symptom burden, and increase access to supportive care resources among Latina breast cancer patients.

**International Registered Report Identifier (IRRID):**

PRR1-10.2196/14339

## Introduction

### Background

Among Latinas/Hispanics (henceforth referred to as Latinas), breast cancer is the most commonly diagnosed cancer and the leading cause of cancer-related deaths [1]. Compared with non-Latina breast cancer survivors (BCSs), Latina BCSs report multiple disparities including poorer health-related quality of life (HRQoL), greater symptom burden, greater cancer-related psychosocial needs [2-4], less breast cancer knowledge, and more dissatisfaction with information related to breast cancer care [5]. Prior studies show that poorer HRQoL is related to lower levels of adherence to disease surveillance and adjuvant treatment [6,7], and interventions designed to improve HRQoL specifically for Latina BCSs can improve adherence to posttreatment care and health outcomes [2,8,9]. However, given the documented disparities beyond HRQoL, interventions targeting Latina BCSs must also provide education about cancer-related symptoms and evidence-based tools to manage symptoms and associated distress.

### Objectives

To address poor HRQoL among Latina BCSs, our team previously developed and pilot tested *My Guide,* a smartphone-based app designed specifically for Latina BCSs to improve HRQoL and reduce symptom burden after completing primary breast cancer treatment [10]. The initial *My Guide* app is available in both English and Spanish, expanding its reach to underserved Latina BCSs. Preliminary findings show that *My Guide* is feasible for Latina BCSs to use (eg, acceptable app usage and high user satisfaction) [11]. However, many of our participants noted that they wished that they had access to the app content *during* treatment.

To date, few interventions have been developed to meet the needs of Latina women undergoing active treatment for breast cancer [12]. There is a critical need for culturally informed interventions designed to address the supportive care needs of Latina women in active treatment, particularly for primarily Spanish-speaking women [3]. Given the high rates of internet and smartphone use documented among US Latinos [13], electronic health–based interventions may capitalize on opportunities to provide scalable resources to Latinas undergoing active treatment while limiting participant burden [14-17].

In light of the limited technology-assisted interventions specifically designed for Latina women undergoing active treatment for breast cancer, more studies are needed to establish the feasibility of smartphone-based interventions for Latinas during active cancer treatment [12]. In response to the feedback from our participants enrolled in the *My Guide* pilot study and the dearth of literature regarding interventions for Latina breast cancer patients actively undergoing treatment, we have developed *My Guide for Breast Cancer Treatment*. The purpose of this paper is to describe the procedures and methods of this follow-up intervention. Guided by Bowen et al’s conceptual model of feasibility studies [18], we describe the plans for conducting a multisite, randomized controlled trial (RCT) to establish the feasibility of *My Guide for Breast Cancer Treatment* for Latina women undergoing breast cancer treatment.

## Methods

### Hypotheses

We hypothesize that Latina women will find *My Guide for Breast Cancer Treatment* feasible for accessing information related to breast cancer and its treatment and for learning strategies to improve symptom management, self-efficacy, communication, and stress. We also hypothesize that women randomized to the *My Guide for Breast Cancer Treatment* app will have better primary outcomes (ie, HRQoL) and secondary outcomes (ie, symptom burden, anxiety, depressive symptoms, and distress) than women randomized to an enhanced usual care control condition.

### Study Design

This RCT is designed to assess the preliminary feasibility and efficacy of the *My Guide for Breast Cancer Treatment* smartphone app for improving HRQoL and symptom burden in Latina women receiving treatment for primary breast cancer. After providing informed consent, participants will be randomized to the *My Guide for Breast Cancer Treatment* intervention condition or to the enhanced usual care control condition. Randomization will be stratified by recruitment site and language (ie, English and Spanish). Participants randomized to the *My Guide for Breast Cancer Treatment* intervention condition will have access to the smartphone app for 12 weeks and will be encouraged to use the app for 1.5 hours each week. The *My Guide for Breast Cancer Treatment* app is accessible on all smartphones (eg, Apple and Samsung) as well as tablets and computer websites. It is available in both English and Spanish, and participants will be provided the app in their preferred language. Participants randomized to the *My Guide for Breast Cancer Treatment* intervention condition will be oriented to the app and instructed to use it for 1.5 hours each week for a duration of 12 weeks. A 12-week intervention timeframe was selected based on previous studies indicating benefits in HRQoL and symptom burden outcomes after 10 weeks of behavioral interventions and the expected length of adjuvant treatments [19-21]. Thus, we believe that 12 weeks will allow sufficient time for participants to access and benefit from the *My Guide for Breast Cancer Treatment* app content. Intervention participants will also be assigned a bilingual telecoach for the duration of the study. Using a stepped-care approach [22], intervention participants will receive weekly telecoaching calls based on their level of adherence to the recommended app usage.

Study outcomes and intervention targets will be measured at study enrollment and before randomization (T1), 6 weeks postbaseline (T2), and 12 weeks postbaseline (T3). All assessment time points will consist of measures assessing our primary outcomes, intervention targets, and secondary outcomes. In addition, all participants will complete a sociodemographic questionnaire at T1, and participants randomized to the intervention condition will be asked to complete an exit survey at T3. Assessments are estimated to take approximately 35 min to complete. Participants randomized to the intervention condition will also be asked to complete brief, 3-min, weekly questionnaires throughout the course of the 12-week intervention. See section *Data Collection and Outcomes* for greater detail. Participants will be compensated US $100 for their participation in the study and receive partial reimbursement for telephone data usage plans. Participants without an internet-enabled device will be provided one for the duration of the study. All study procedures and assessments have been approved by the institutional review board (IRB) of record for this multisite study (Northwestern IRB).

### Participants

Participants will be 60 Latina women who have been diagnosed with stage I-IIIA breast cancer, have completed surgery (eg, mastectomy and lumpectomy), and are actively receiving adjuvant treatment for breast cancer (eg, chemotherapy, radiation, and biologic therapy). We will enroll participants from the Robert H. Lurie Comprehensive Cancer Center at Northwestern Memorial Hospital, the University of Illinois Health System, and various community-based support groups sponsored by ALAS-WINGS, a Chicago-based nonprofit organization for Latina breast cancer patients.

### Eligibility

We will identify participants through screening of the electronic medical record (EMR), and final determinations for study eligibility will be made based on a self-report questionnaire via telephone interview. To be included in the study, participants must (1) be female, because male breast cancer patients represent less than 1% of the breast cancer cases; (2) have a diagnosis of stage I-IIIA breast cancer; (3) have completed surgery for breast cancer; (4) be within any timepoint of the adjuvant treatment trajectory for breast cancer (eg, chemotherapy, radiation, surgery, and biologic therapy); (5) be at least 18 years of age; (6) be able to speak and read English or Spanish; (7) be able to provide informed consent; and (8) self-identify as Hispanic/Latina ethnicity. Participants will be excluded from the study if they (1) have a hearing, motor, visual, or voice impairment that would preclude completion of study procedures; (2) have been diagnosed with a psychotic disorder, bipolar disorder, dissociative disorder, or any other diagnosis for which study participation would be inappropriate or dangerous; (3) endorse suicidal ideation, plan, or intent; (4) endorse illicit substance or alcohol dependence; or (5) have been diagnosed with dementia. Patients with metastatic disease will not be included in this study, as they have more extensive treatment regimens and substantially different needs relative to patients with earlier stages of breast cancer.

### Randomization

Eligible participants who provide informed consent will be individually randomized using a 1:1 ratio to either the intervention condition (*My Guide for Breast Cancer Treatment*) or the control condition (enhanced usual care) for a total of 3 months.

### Study Conditions and Delivery

#### My Guide for Breast Cancer Treatment Development and Content

*My Guide* was initially designed to improve HRQoL and reduce symptom burden among Latina BCSs (ie, BCSs who have completed active treatment) [10]. *My Guide for Breast Cancer Treatment* has been adapted from *My Guide* to expand its reach to Latina women in active treatment for breast cancer. The *My Guide* app incorporates elements in the published literature on symptom management in breast cancer literature and investigators with ample clinical expertise and experience conducting behavioral and symptom management research with Hispanic BCSs. The content for both iterations of the intervention were informed by models of stress and coping, cognitive behavioral stress management [23-25], the extant literature related to psychosocial adaptation to breast cancer [26-28], and studies evaluating these models and skills among Latina BCSs [8,9,26,27,29-31]. *My Guide* and *My Guide for Breast Cancer Treatment* are culturally informed by Latina values and beliefs including familism, fatalism, external locus of control, and culturally shaped gender roles (ie, Machismo and Marianismo) [30,32,33]. In addition, they both address challenges that may disproportionally affect Latina women, such as language barriers and concerns related to citizenship. In addition, to address low literacy concerns, all the intervention content is accessible through an audio format that is embedded within each app.

*My Guide for Breast Cancer Treatment* expands the scope and focus of the intervention by including additional content specifically for women in active treatment. In addition, *My Guide for Breast Cancer Treatment* includes content related to diet, physical activity, and general health information for breast cancer patients. The *My Guide for Breast Cancer Treatment* intervention content is organized into 6 modules as described in Table 1, which include content related to breast cancer education (eg, disease-related information and common breast cancer treatments); common physical, psychological, and emotional symptoms women may experience during and after breast cancer treatment; changes to daily life (eg, responsibilities at work and home and relationships with close family and friends); and local community resources for Latinas with breast cancer. In addition, for easy reference, videos and audio recordings from all the sections are listed and organized by topic in the last module (*Listen and Learn*).

**Figure 1:**
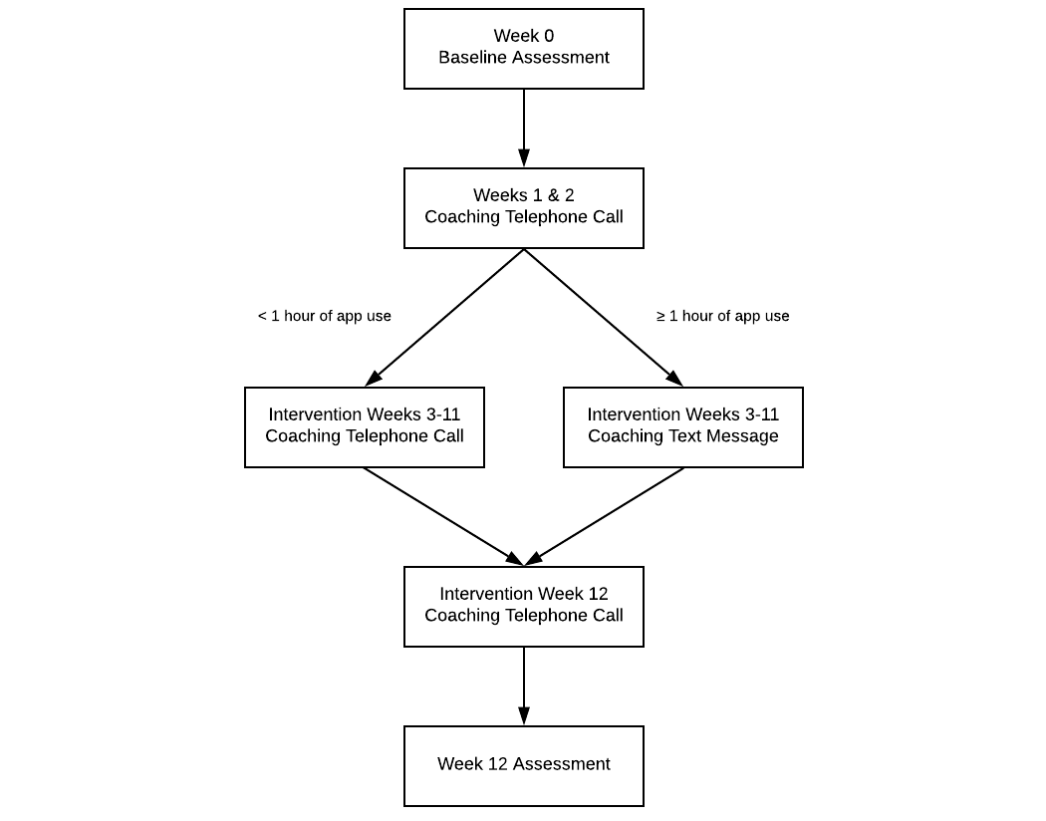
Telecoaching stepped-care protocol.

All intervention participants will be assigned a trained, bilingual telecoach who will provide telecoaching using a stepped-care approach (Figure 1) to enhance adherence to the recommended *My Guide for Breast Cancer Treatment* app usage. In a given week, participants who use the app for less than 1 hour will be considered nonadherent to the recommended app usage and will receive a telecoaching call. Participants who use the app for 1 hour or more will be considered adherent to the recommended usage and will not receive a telecoaching call. Rather, adherent participants will receive a reinforcing SMS text message. The 1-hour cut-off was based on data from the previous *My Guide* trial for cancer survivors in which participants’ average use of the study app exceeded 1 hour per week [10].

**Table 1:** *My Guide for Breast Cancer Treatment* main content modules.

Module	Description of information provided	Examples
Managing My Symptoms	Common physical and psychological symptoms patients experience during and after cancer treatment	Nausea and vomiting, fatigue, lymphedema, sadness, worry
Managing My Emotions	Emotions commonly experienced during and after cancer treatment	Anger, anxiety, sadness/depression; improving thoughts and feelings; expressing your feelings
Understanding Your Breast Cancer	Breast cancer, adjuvant cancer treatments and related side effects	Breast cancer and treatments overview; following doctor recommendations
Living Well After Treatment	Maintaining a healthy lifestyle during treatment	Diet and physical activity content specific for patients with cancer
Friends and Family	Changes in roles and responsibilities at home and work, relationships with family and friends, and daily activities during and after cancer treatment	Changes to relationships, sexual/intimacy, and at work; advice for singles; talking to your doctor
Community and Everyday Support	Local community organizations and resources for Latinas in the Chicagoland area for additional support	Spanish support groups, transportation assistance, financial assistance

The telecoaching calls will be brief (15 min) and focus on encouraging adherence to *My Guide for Breast Cancer Treatment* using the principles of motivational interviewing [34]. During the first call, participants will complete a decisional balance exercise with their telecoach to identify and address potential ambivalence about using the app for the recommended time. Guided by the telecoach, participants will set weekly goals regarding their app usage for the upcoming week, which will be reviewed in subsequent telecoaching calls. A central topic of the telecoaching calls will be barriers and facilitators to app usage, allowing telecoaches to problem solve with participants and set future app usage goals. Importantly, the goal of telecoaching calls is not to deliver intervention content but rather to facilitate adherence to the *My Guide for Breast Cancer Treatment* app.

All telecoaches will receive training in motivational interviewing and goal setting with a particular focus on sensitivity to issues relevant for Latina women (eg, cultural beliefs that may influence coping and health behaviors). Telecoaches will have access to participants’ app usage through a study administrative site. All telecoaching calls will be audio-recorded, and approximately 20% of the telecoaching calls will be reviewed weekly in supervision with a licensed clinical psychologist to monitor intervention fidelity.

One of the most significant additions to the intervention app is that *My Guide for Breast Cancer Treatment* will be individually tailored to each participant’s self-reported primary concerns/symptoms. Upon randomization, participants in the intervention condition will complete a questionnaire based on the MD Anderson Symptom Inventory [35] to assess for common breast cancer and breast cancer treatment–related concerns, complaints, and symptoms. *My Guide for Breast Cancer Treatment* will then organize the app presentation such that content related to each participant’s 3 to 5 top-rated concerns/symptoms are most easily accessible in a separate tab titled “Just for Me*.*” Participants will complete this questionnaire every 2 weeks, with subsequent content-related changes made to their “Just for Me” tab. As tailored information is added to this tab, the most recent additions will appear at the top of this tab, and older content will move to the bottom. Notably, participants will still have access to all of the intervention content. However, this adaptive feature will help participants focus on and prioritize their primary concerns and needs.

Each week participants will be incrementally rewarded/reinforced for the time spent using the *My Guide for Breast Cancer Treatment* app. Gamification is use of game-design elements to engage and motivate individuals to achieve a goal [18]. Although the gamification features are becoming increasingly popular in behavioral and medical interventions, only a few studies have demonstrated their potential effectiveness [36-38]. *My Guide for Breast Cancer Treatment* contains 3 levels of virtual reinforcement in a given week. Participants may receive a virtual ribbon after completing 30 min of app use, a virtual medal after completing 45 min of app use, and a virtual trophy after completing 60 min of app use. Therefore, participants who use the app for a full hour each week for the length of the study can receive a total of 12 ribbons, 12 medals, and 12 trophies [39].

**Figure 2:**
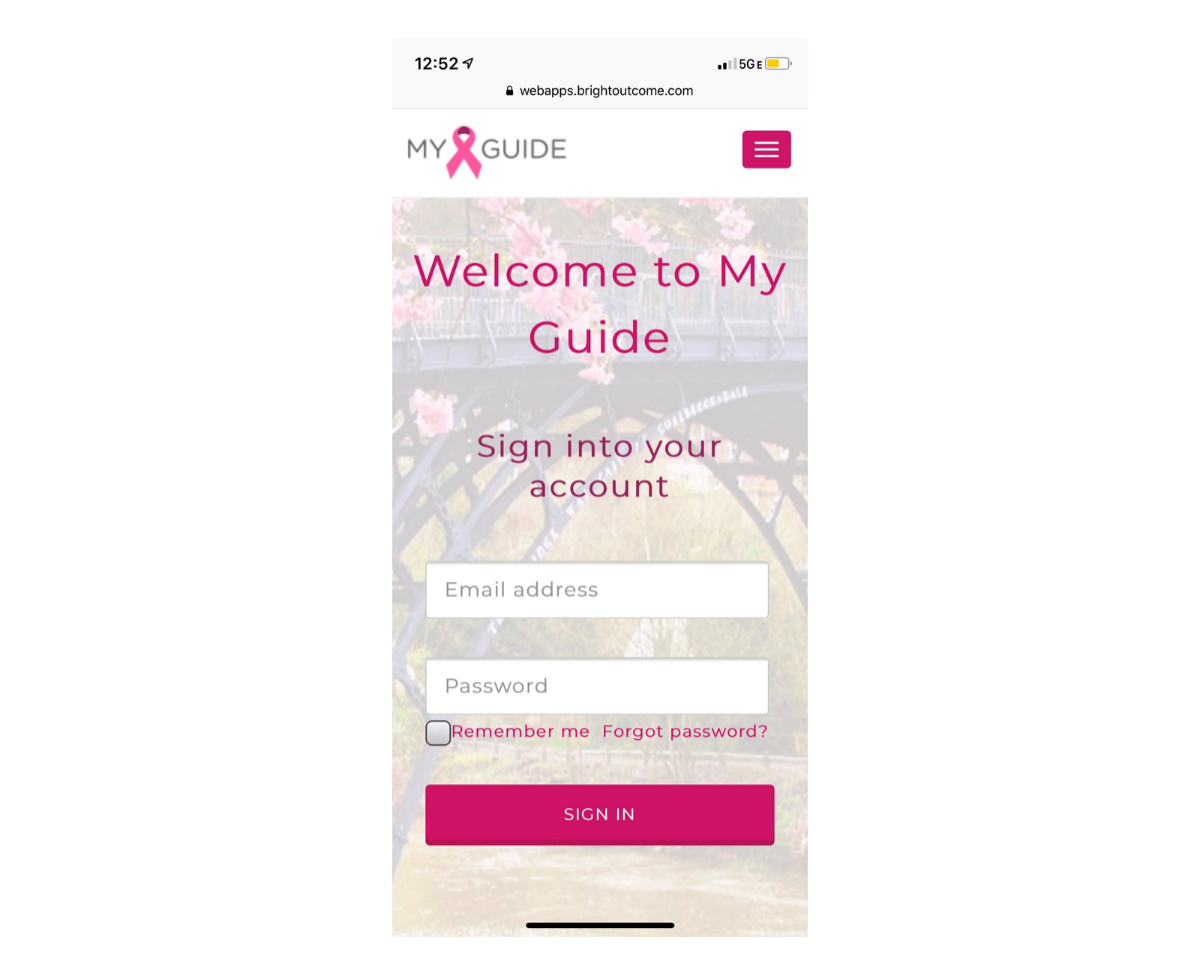
Screenshot of the *My Guide* smartphone app.

The smartphone app for the *My Guide for Breast Cancer Treatment* intervention condition is currently in development with Bright Outcomes Company (see Figure 2).

Participants will be able to securely log in to the app, and participant usage and engagement with video/audio features will be captured and sent to a secure and Health Insurance Portability and Accountability Act–compliant medical school server housed at Bright Outcomes. Security measures to protect privacy threats associated with users’ computers and devices include the following measures: users are automatically logged out of intervention and assessment tools after 20 min of inactivity; any data stored locally are automatically encrypted based on the user authentication information and cannot be accessed without this information; all information collected by the app will be immediately transmitted to the secure server; and no app data will be saved to the phone’s memory.

Participants randomized to the enhanced usual care control condition will receive care as usual and printed educational materials from the National Cancer Institute related to breast cancer and survivorship [40], patient education in either English or Spanish, as well as a list of Chicago-based supportive care resources and organizations. They will not receive a smartphone app or telecoaching during the study. Participants randomized to the control condition will be given access to the *My Guide for Breast Cancer Treatment* app after completing the T3 assessment, a period of 3 months.

### Data Collection and Outcomes

Consistent with past research, the feasibility of *My Guide for Breast Cancer Treatment* intervention will be evaluated by assessing the acceptability and demand of the app [18]. Acceptability of the app will be assessed at T3 using a previously tested app satisfaction questionnaire [20] that measures the usefulness, satisfaction, learnability, and usability of *My Guide for Breast Cancer Treatment.* Above average scores will be considered acceptable. Demand will be measured with study recruitment, retention, and participant use of *My Guide for Breast Cancer Treatment* (eg, frequency of log-ins, time spent using the app, and content accessed). Recruitment and retention rates of 70% or more will be considered acceptable based on our prior work and published studies [20,41,42].

All measures will be administered in each participant’s preferred language. For assessments completed in Spanish, we will use measures that have either been previously translated to Spanish and validated or measures that were translated to Spanish for this study by IRB-approved translators.

Participants will complete a self-report sociodemographic questionnaire at T1 including questions related to age, racial background, Latina ancestry, income, employment, relationship status, educational attainment, years living in the United States, and subjective social status using the MacArthur scale [43]. Participants will self-report clinical information including time since breast cancer diagnosis and surgery, stage of diagnosis, type of surgery, current treatment regimen (eg, chemotherapy and radiation), additional medications, medical comorbidities (assessed using the Charlson Comorbidity Index [44]), and past or current psychiatric diagnoses. Financial toxicity will be measured using Comprehensive Score for financial Toxicity-Functional Assessment of Chronic Illness Therapy [45]. All self-reported medical- and disease-related information will be verified via medical chart review.

#### Health-Related Quality of Life

At T1 to T3, participants will complete the Functional Assessment of Cancer Therapy-Breast (FACT-B). The FACT-B has been extensively used among breast cancer patients [46,47] and measures physical, emotional, social, and functional well-being, as well as breast cancer–related concerns over the past 7 days using a 5-point response scale [46]. Participants randomized to the *My Guide for Breast Cancer Treatment* intervention condition will also complete the rapid version of the Functional Assessment of Cancer Therapy-General (FACT-G7) on their smartphones every week throughout the 12-week intervention. The FACT-G7 assesses the most prominent HRQoL concerns among cancer patients and is both valid and reliable [48]. The FACT-G7 takes approximately 5 min to complete and is therefore an ideal brief measure of HRQoL to include in a smartphone-delivered intervention.

#### Symptom Burden

At T1 to T3, participants will complete the Breast Cancer Prevention Trial (BCPT) symptom questionnaire. The BCPT is a 25-item questionnaire that asks participants to use a 5-point response scale to rate their level of discomfort with common breast cancer–related symptoms during the past 4 weeks [49].

#### Anxiety and Depression

Anxiety and depressive symptoms will be measured at T1 to T3 using the Patient-Reported Outcomes Measurement Information System (PROMIS) [50,51] via brief computer adaptive tests (CATs). With CATs, participants’ responses guide the system’s choice of subsequent items from a bank of 29 items for anxiety and 28 items for depression.

#### Fear of Cancer Recurrence

At T1 to T3, participants will complete the Concerns About Recurrence Scale to assess fears about breast cancer recurrence [52]. Items address numerous life domains that could be impacted by a breast cancer recurrence, such as physical health and relationships. Participants are asked to use a 5-point response scale to indicate the extent to which they felt threatened by a recurrence in each domain.

#### Physical Activity

Physical activity will be measured at T1 to T3 using the International Physical Activity Questionnaire (IPAQ)-short form [53,54]. This measure consists of 7 items that assess physical activity over the past 7 days, and participants record the number of days per week or minutes per day spent doing specific activities. Previous reviews have demonstrated that the IPAQ-short form has high reliability [55,56], and the test-retest reliability of the questionnaire was at an acceptable level [55].

#### Dietary Intake

Dietary intake will be measured at T1 to T3 using the Brief Dietary Assessment Tool for Latinas [57]. This screening tool assesses fruit, vegetable, and fat intake over the past month.

#### Self-Efficacy

A total of 3 self-report questionnaires will be used to measure cancer-relevant self-efficacy at T1 to T3. First, the Communication and Attitudinal Self-Efficacy scale for cancer (CASE-cancer) is a psychometrically valid 12-item measure. A total of 2 CASE-cancer subscales will be used to assess a person with cancer’s self-efficacy in communication and information seeking [58]. Participants rate their level of confidence related to 12 skills using a 4-point response scale. This measure has been previously used with Latina BCSs [58,59]. Second, the PROMIS Self-Efficacy for Managing Emotions CAT will be administered, which assesses a person’s level of confidence to (1) manage symptoms of anger, anxiety, depression, disappointment, discouragement, frustration, and helplessness and (2) prevent symptoms from interfering with daily activities [60]. This measure has an item bank of 25 items. Finally, the 3-item “Assertiveness” scale from the Measure of Current Status will be used to assess a participant’s degree of confidence about asking and expressing their needs [61]. 

#### Breast Cancer Treatment Knowledge

The Knowledge about Breast Cancer questionnaire will be used to assess knowledge related to treatment at T1 to T3. This scale consists of 16 true or false statements regarding general breast cancer knowledge. The average number of correct responses will be calculated. This questionnaire was previously tested with a large sample of Spanish-speaking Latina BCSs [62] and used in the initial *My Guide* pilot study [10].

#### Cancer-Specific Distress

The Impact of Events Scale will be used to assess cancer-specific distress at T1 to T3. This measure comprises 2 subscales assessing the frequency of intrusive thoughts and avoidance following a stressful event [63,64].

### Analytic Plan

Descriptive statistics will be used to characterize the overall sample, each condition, and study acceptability and demand. To examine preliminary differences in feasibility between study conditions, we will use the chi-square test, Fisher exact test, and nonparametric Mann-Whitney *U* test for non-normal data, as appropriate. With 30 participants per study condition, there will be 80% power to detect approximately one-half SDs, assuming a 2-tailed test and a type I error rate of 5%. All power calculations were run using PROC POWER in SAS version 9.4.

For each study outcome, average changes from pre intervention to post intervention will be calculated via means (SD) and 95% CIs, and the mean changes within study condition will be converted to effect sizes. An analysis of covariance approach will be used to examine differences between the intervention and control conditions in follow-up scores, after adjusting for baseline score. Established cut-offs will also be referenced for clinically meaningful differences for the FACT-B and FACT-B subscales to identify ranges and patterns of changes in scores across conditions from baseline to follow-up.

### Data Management

All study data will be managed and stored in Research Electronic Data Capture (REDCap), a secure Web-based research data management system hosted at Northwestern University. All IRB-approved research team members will have access to REDCap. REDCap will be used to organize EMR data and to administer all study questionnaires. The *My Guide for Breast Cancer Treatment* administrative interface will capture participants’ app usage and weekly questionnaire data, which will be uploaded to REDCap to centralize all research information. Once all data are collected, the data will be imported into SPSS for data cleaning and analysis.

## Results

Recruitment began in July 2019 and is expected to be completed by August 2020. We expect to submit study results for publication by fall 2020.

## Discussion

### Overview

The aim of the *My Guide for Breast Cancer Treatment* study is to improve HRQoL and reduce symptom burden among Latina women undergoing active treatment for breast cancer. To the best of our knowledge, this is the first bilingual, smartphone-based supportive care app for Latinas women with breast cancer in active treatment. Providing a supportive and behavioral intervention that focuses on cancer treatment education and self-management during active treatment has the potential to improve patient-reported outcomes and clinical outcomes during active treatment and into survivorship.

Compared with the first version of the *My Guide* smartphone app, our team implemented substantial changes in this version of the *My Guide for Breast Cancer Treatment* app. Adding an adaptive functionality offers *My Guide for Breast Cancer Treatment* users a more personalized approach to delivering the intervention content, and the addition of a gamification via virtual ribbons, medals, and trophies provides users with encouragement to sustain use of the app across the length of the study. Unlike our first *My Guide* study [10] that included a 2-month longitudinal trial, we have extended the time frame of this study to 3 months to accommodate the expected time frame during which our study participants will be receiving adjuvant treatment. These strategic changes make this version of the *My Guide for Breast Cancer Treatment* app and this study design better suited for women in active treatment.

### Limitations

A few limitations of the study protocol warrant discussion. First, because the focus of this study is on breast cancer patients who are in active treatment, long-term cancer survivors will not be enrolled in this study, which limits study generalizability. In addition, women who are preparing for surgery will not be included in this study. However, *My Guide for Breast Cancer Treatment* does include a focus on the postsurgical recovery phase such as information related to postsurgical symptoms (eg, pain and lymphedema) and information related to reconstructive surgery.

### Future Directions

There are several areas for future directions. If found feasible, future studies should establish the efficacy of *My Guide for Breast Cancer Treatment* across a nation-wide, heterogeneous sample of Latina women diagnosed with breast cancer. Currently, the *My Guide for Breast Cancer Treatment* app does not include any social media aspects; the potential benefit of adding a social media component to *My Guide for Breast Cancer Treatment* may be another important direction for future iterations of the app. In addition, given the multiple components of this feasibility trial (ie, app, telecoaching, and SMS text messaging), another important future direction will be to disentangle the effects of each component of the intervention on study outcomes.

Findings from this second study of the *My Guide for Breast Cancer Treatment* app are expected to contribute to the literature by establishing the efficacy of a smartphone-delivered intervention to enhance self-management during breast cancer treatment for improving HRQoL and symptom burden among Latina patients. Given the technology-based delivery of this app, the *My Guide for Breast Cancer Treatment* app has the potential for nation-wide scalability and therefore can increase access to supportive care resources among Latina breast cancer patients.

## Appendix

Multimedia Appendix 1 Peer-reviewer report from the Chicago Cancer Health Equity Collaborative.

## Abbreviations

BCS: breast cancer survivor
BCPT: Breast Cancer Prevention Trial
CASE-cancer: Communication and Attitudinal Self-Efficacy scale for cancer
CAT: computer adaptive test
EMR: electronic medical record
FACT-B: Functional Assessment of Cancer Therapy-Breast
FACT-G7: Functional Assessment of Cancer Therapy-General
HRQoL: health-related quality of life
IRB: institutional review board
IPAQ: International Physical Activity Questionnaire
PROMIS: Patient-Reported Outcomes Measurement Information System
RCT: randomized controlled trial
REDCap: Research Electronic Data Capture
