# Supplementary material for: An Electronic Health Intervention for Latina Women Undergoing Breast Cancer Treatment (My Guide for Breast Cancer Treatment): Protocol for a Randomized Controlled Trial
Source: JMIR Res Protoc. 2019 Dec 13;8(12):e14339. doi: 10.2196/14339 (PMC6935046; doi:10.2196/14339)
Supplement: Multimedia Appendix 1 [file resprot_v8i12e14339_app1.pdf]

ChicagoCHEC Incubator & Catalyst Grants Mechanism: Full Research Project

Application #: 01

Application Title: The My Guide e-Health Intervention for Latina/Hispanic Women Receiving Treatment for Breast Cancer (Short Title: My Guide)

Principal Investigator(s): Betina Yanez (NU), Frank Penado (NU), Alejandra Perez-Tamayo (UIC) Joanna Buscemi (UIC), Francisco Iacobelli (NEIU), Judy Guitelman (ALAS-Wings)

Cancer focus (select all that apply):

- ▫ Basic science
- ▫ X Clinical
- ▫ Translational
- ▫ Prevention
- ▫ X Control
- ▫ X Behavioral
- ▫ X Population research
- ▫ Policy

CONFIDENTIAL

OVERALL IMPACT

Reviewers will provide an overall impact score to reflect their assessment of the likelihood for the project to exert a sustained, powerful influence on the research field(s) involved, in consideration of the following nine scored review criteria, and additional review criteria. An application does not need to be strong in all categories to be judged likely to have major scientific impact.

Overall Impact *Write a paragraph summarizing the factors that informed your Overall Impact score.*
**Overall Impact Score: __2_______** This project represents an important study in the field and addresses a research gap on cancer survivorship in Latinas. The application has a high likelihood of getting external funding and the approach and rigor and early outcomes indicate a high likelihood for the project to advance to receive external.

SCORED REVIEW CRITERIA

Reviewers will consider each of the nine review criteria below in the determination of scientific and technical merit, as well as relevance to the partnership goals and objectives, and give a separate score for each (9-point scale). For NIH guidelines on scoring using a 9-point scale, please review the NIH guidelines:

"Scoring System and Procedure"
http://grants.nih.gov/grants/peer/guidelines_general/scoring_system_and_procedure.pdf

"Interpreting new application scores and critiques"
http://enhancing-peer-review.nih.gov/docs/scoring_and_critique_overview_June2009.pdf

1. **Significance.** The project addresses important problems or critical barriers to progress in cancer research or cancer disparities research. The aims of the project advance scientific knowledge, technical capability, and have near-term impact.

Significance Score: ____1_____

Strengths

- **Addresses a key areas in population health and survivorship research**
- **Addresses key cultural implications to examine and improve outcomes in Latina breast cancer patients**
- **Has impact that can be tailored and generalized to other cultural groups for survivorship**

Weaknesses

- None

2. **Innovation.** The application utilizes novel theoretical concepts, approaches or methodologies, instrumentation, or interventions.

Innovation Score: ___1______

Strengths

- **The use of technology based approach in this topic is innovative and novel**
- **The user derived approach to develop the application helps to ensure the approach is community and patient centric**
- **The culturally tailored approach is innovative and the use of Spanish speaking patients is key**

CONFIDENTIAL

Weaknesses

- Minor weakness but more information is needed about the broad use of smartphones and availability of smartphones in the Latino community specifically among the targeted age group

3. **Investigators.** The PIs, collaborators, and other key personnel have the necessary experience and expertise to accomplish the goals of the proposed research project. The investigators have complementary and integrated expertise.

Investigators Score: _1________

Strengths

- This is a strong multi-disciplinary team
- The use of social scientists, behavioral scientists and clinician scientist is a very strong approach to the multi-investigator team
- The engagement of the community partner is important and key to sustainability

Weaknesses

- One minor weakness may be the little research experience of the clinician scientist, but it is minor and her role provides a key focus of how to engage clinicians in cancer survivorship research.

4. **Approach** The overall strategy, methodology, and analyses is well-reasoned and appropriate to accomplish the specific aims of the project.

Approach Score: ____2_____

Strengths

- The methods are well developed to achieve the study aims
- The approach to first test the feasibility in a pilot manner is a good approach

Weaknesses

- Little information about the theoretical framework that guides the approach

5. **Career Development** There is a well thought-out and reasonable plan for career development of junior investigator(s). Mentor(s) have been identified and the appropriate mechanisms are in place to achieve career development goals outlined.

Career Development Score: _____1____

Strengths

- Strong career development plan for Dr. Yanez

CONFIDENTIAL

- Good description of how the mentors will be utilized
- Strong early outcomes in publications and conferences and grant submission of the PI

Weaknesses

- Little discussion on the career development of the NEIU partners
- Little discussion on the career development of the community partner. How does this project benefit the community partner's priority?

6. **Community Engagement**. The partner organization(s) and their respective roles and contributions are clearly articulated.

Community Engagement Score: ___2______

Strengths

- Good use of engagement of the community partner
- The use of UIC investigator is a good use of academic community partner

Weaknesses

- Little discussion on dissemination plan
- Great that the community partners will invite key stakeholders to attend weekly meetings, but need more discussion on outcomes of these weekly meetings

7. **Training**. There will be significant opportunity for the training and research experiences for students and early stage investigators underrepresented in biomedical and cancer research

Training Score: ___2______

Strengths

- Strong opportunity to advance training of research team and ESI
- Great early demonstrations of how the project is advancing the professional development of the ESI

Weaknesses

- Little focus on research training of RAs and students h

8. **Potential to Generate Usable Data**. There is high likelihood that the proposed project or program will generate usable data, including preliminary data for future grant proposals or data for peer-reviewed publications

Potential Data Score: ______1___

CONFIDENTIAL

**Strengths**<br>• Great early outcomes data that will provide key information for future grant proposals<br>• Great dissemination of preliminary data to research community<br>**Weaknesses**<br>• Minor concern on lack of data on how Latinas use mobile devices

9. **Potential for External Funding**. There is high likelihood that the proposed project or program will lead to more competitive grant applications to NIH and other types of foundation funding
**Potential Funding Score: ____1_____**<br><br>**Strengths**<br>• This application has a VERY high likelihood of getting external funding<br>• The early publication and presentation history of the investigator team provides early outcome data<br>**Weaknesses**<br>• None

CONFIDENTIAL

ADDITIONAL REVIEW CONSIDERATIONS

As applicable for the project proposed, reviewers will address each of the following items, but will not give scores for these items and should not consider them in providing an overall impact/priority score.

Budget and Period of Support
Click Here to Select<br>Recommended budget modifications or possible overlap identified:<br>•

ADDITIONAL COMMENTS TO APPLICANT

Reviewers may provide guidance to the applicant or recommend against resubmission without fundamental revision.

Additional Comments to Applicant (Optional)
•

Community Partner

ChicagoCHEC Incubator & Catalyst Grants Mechanism: Full Research Project

Application #: 01

Application Title: The My Guide e-Health Intervention for Latina/Hispanic Women Receiving Treatment for Breast Cancer (Short Title: My Guide)

Principal Investigator(s): Betina Yanez (NU), Frank Penado (NU), Alejandra Perez-Tamayo (UIC) Joanna Buscemi (UIC), Francisco Iacobelli (NEIU), Judy Guitelman (ALAS-Wings)

Cancer focus (select all that apply):

- ◻ Basic science
- ◻ Clinical
- ◻ Translational
- ◻ Prevention
- ◻ Control
- ◻ Behavioral
- ◻ Population research
- ◻ Policy

CONFIDENTIAL

OVERALL IMPACT

Reviewers will provide an overall impact score to reflect their assessment of the likelihood for the project to exert a sustained, powerful influence on the research field(s) involved, in consideration of the following nine scored review criteria, and additional review criteria. An application does not need to be strong in all categories to be judged likely to have major scientific impact.

Overall Impact *Write a paragraph summarizing the factors that informed your Overall Impact score.*
**Overall Impact Score: __1_______**<br><br>**By creating innovative and interdisciplinary pathways to access to bilingual care and education, I believe "My Guide" will have a powerful and sustained influence on the research fields involved because of its robust capacity to reduce disparities for Latina women diagnosed with breast cancer. The outcomes will cultivate new standards for quality of life for Latina women that will have an intergenerational impact on patients' mothers and children, further strengthening family bonds and community bonds. In turn, this project can catalyze the Latina community to be proactive and active with their health and also foster a climate of optimism and hope for the whole community. While validating**

and addressing culturally and linguistically specific barriers that exist, the project provides mechanisms, tools, education, and care for the community to participate in the solution to the high rate of breast cancer in Latina women. The professional credentials of the principal investigators, innovative and interdisciplinary design across all research fields involved, the comprehensive implementation and utilization of the data generated from the earlier pilot program, the focus on an underrepresented minority that suffers from a high rate of breast cancer, the unique integration and application of Latina/Hispanic culture to build a linguistically and culturally tailored project available in two languages, the innovative way it addresses quality of life issues including preventative care, psychosocial survivorship issues, the utilization and application of cutting edge AI technology to develop new bilingual tools for patients, and the opportunities for the principals to advance the scientific field by creating opportunities for students, mentors, publishable data and publishing opportunities in peer-reviewed publications. The mechanisms, tools, and methodologies created will contribute to an ostensible shift in the Latina psychosocial climate because this project thematically addresses the role of alienation and anxiety in cancer.

CONFIDENTIAL

SCORED REVIEW CRITERIA

Reviewers will consider each of the nine review criteria below in the determination of scientific and technical merit, as well as relevance to the partnership goals and objectives, and give a separate score for each (9-point scale). For NIH guidelines on scoring using a 9-point scale, please review the NIH guidelines:

"Scoring System and Procedure"
http://grants.nih.gov/grants/peer/guidelines_general/scoring_system_and_procedure.pdf

"Interpreting new application scores and critiques"
http://enhancing-peer-review.nih.gov/docs/scoring_and_critique_overview_June2009.pdf

1. **Significance.** The project addresses important problems or critical barriers to progress in cancer research or cancer disparities research. The aims of the project advance scientific knowledge, technical capability, and have near-term impact.

Significance Score: ___1______

Strengths

- **Advanced development with technological applications that will directly serve Latina patients in their care and post-care, fostering access and support, Also, this helps instill faith and trust in the medical community and helps the community bonds strengthen through new shared experiences.**

Weaknesses

-

2. **Innovation.** The application utilizes novel theoretical concepts, approaches or methodologies, instrumentation, or interventions.

Innovation Score: ____1_____

Strengths

- **Bilingual and culturally tailored designs, application of novel theoretical concepts and methodologies, interinstitutional team, high quality of professional credentials of principals, mechanisms for sustained impact**

Weaknesses

- I wonder if it might be useful to harness the student fellow alumni in future projects.

3. **Investigators.** The PIs, collaborators, and other key personnel have the necessary experience and expertise to accomplish the goals of the proposed research project. The investigators have complementary and integrated expertise.

Investigators Score: _____1____

Strengths The investigators have demonstrated their excellence and expertise in previous CHEC projects and grants.

Weaknesses

-

4. **Approach** The overall strategy, methodology, and analyses is well-reasoned and appropriate to accomplish the specific aims of the project.

Approach Score: ____1_____

Strengths The strategy, methodology and analyses are strongly tied to generating successful outcomes. The role of technology, AI and the app particularly stand out, as well as the attention to self-care and wellness.

-

Weaknesses

-

CONFIDENTIAL

5. **Career Development** There is a well thought-out and reasonable plan for career development of junior investigator(s). Mentor(s) have been identified and the appropriate mechanisms are in place to achieve career development goals outlined.

Career Development Score: 1_________

Strengths

- Pathways for junior investigators to advance are robust and abundant. Also, there are excellent plans in place for mentors engage active roles

Weaknesses

-

6. **Community Engagement**. The partner organization(s) and their respective roles and contributions are clearly articulated.

Community Engagement Score: ____1_____

Strengths The roles and contributions of the community partner organization are very clear. The high quality of previously established partnership with the community partner plays an important role in ensuring continuity in relationships already established.

-

Weaknesses

- I am not sure if this is relevant, but I wonder if other community partners are aware of the participation of the community partner organization, and if that would be helpful for the community partners to be updated somehow even if it is superficial like a short update in a newsletter or on a website? Would active communication in the form of updates, etc. help strengthen the psychosocial climate of the Latina community and build interest in CHEC's mission, activities and opportunities.

7. **Training**. There will be significant opportunity for the training and research experiences for students and early stage investigators underrepresented in biomedical and cancer research

Training Score: ____1_____

Strengths

- There is already an excellent precedent set for this set in the earlier two and half years of CHEC.

Weaknesses

-

CONFIDENTIAL

8. **Potential to Generate Usable Data**. There is high likelihood that the proposed project or program will generate usable data, including preliminary data for future grant proposals or data for peer-reviewed publications

Potential Data Score: _______1__

Strengths

- The novel, innovative, interinstitutional and interdisciplinary components of the project will create usable data for the scientific community and help fuel future grant proposals and peer reviewed publications.

Weaknesses

-

9. **Potential for External Funding**. There is high likelihood that the proposed project or program will lead to more competitive grant applications to NIH and other types of foundation funding

Potential Funding Score: _____1____

Strengths

- The excellent leadership of the team of principals is critically important. The earlier successful precedents set by CHEC projects provide and excellent barometer for future grant applications to NIH and other types of foundation funding. The strength and vitality of the team of extremely competent principals will lead to a dynamic presence in the scientific community.

Weaknesses

-

CONFIDENTIAL

ADDITIONAL REVIEW CONSIDERATIONS

As applicable for the project proposed, reviewers will address each of the following items, but will not give scores for these items and should not consider them in providing an overall impact/priority score.

Budget and Period of Support
Click Here to Select<br>Recommended budget modifications or possible overlap identified:<br>• n/a

ADDITIONAL COMMENTS TO APPLICANT

Reviewers may provide guidance to the applicant or recommend against resubmission without fundamental revision.

Additional Comments to Applicant (Optional)
• It has been truly an honor to be a reviewer. Cancer has played a big role in my life, having lost my mom to breast cancer at 52, choosing BRCA2 surgeries, and unexpectedly facing a discovery of a kidney tumor in a spinal MRI for a separate issue. I am passionate and dedicated to helping create access for minorities to good medical care, education and resources for wellness and selfcare. Addressing the importance of cultural differences- biologically, physiologically, socially, emotionally, historically- is part of the larger mission of medical community to contribute to the health and growth of humanity. By creating and implementing solutions to racial disparities will impact not only the scientific field but the Latina community in a holistic way. This carries the potential to forge new, sustaining and impactful pathways to tailor medicine, education and care for all minorities in our society.

CONFIDENTIAL

ChicagoCHEC Incubator & Catalyst Grants Mechanism: Full Research Project

Application #: 01

Application Title: The My Guide e-Health Intervention for Latina/Hispanic Women Receiving Treatment for Breast Cancer (Short Title: My Guide)

Principal Investigator(s): Betina Yanez (NU), Frank Penado (NU), Alejandra Perez-Tamayo (UIC) Joanna Buscemi (UIC), Francisco Iacobelli (NEIU), Judy Guitelman (ALAS-Wings)

Cancer focus (select all that apply):

- ◻ Basic science
- ◻ Clinical
- ◻ Translational
- ◻ Prevention
- ◻ Control
- ◻ Behavioral
- ◻ Population research
- ◻ Policy

CONFIDENTIAL

OVERALL IMPACT

Reviewers will provide an overall impact score to reflect their assessment of the likelihood for the project to exert a sustained, powerful influence on the research field(s) involved, in consideration of the following nine scored review criteria, and additional review criteria. An application does not need to be strong in all categories to be judged likely to have major scientific impact.

Overall Impact *Write a paragraph summarizing the factors that informed your Overall Impact score.*
**Overall Impact Score: __4_______**<br><br>The proposed project addresses the important problem with the Latino/Hispanic female patients with breast cancer and proposes to improve the current My Guide application that was developed by the same team. The application provides a clear goal of adjusting the target population to the active patients by adding new content of the diet and physical activity, as well as individually tailored content to each woman’s phase of treatment and self-reported symptoms and concerns. The proposed trail study is also well organized to serve this goal. It provides great training opportunities to the students and junior faculties, and great opportunities to serve the community.<br><br>The current My Guide application seems well established. The adjustment on the target population is significant, but besides the proposed new content to serve the purpose, the proposal

did not provide more details about the adjustment to the current content to adapt to the change of target population. The role of the psychologists was also not clear for these adjustments since the new contents proposed seems irrelevant to the psychology perspective. The propose did not provide details about the method to compare the current and the proposed application for the significance of the adjustments.

SCORED REVIEW CRITERIA

Reviewers will consider each of the nine review criteria below in the determination of scientific and technical merit, as well as relevance to the partnership goals and objectives, and give a separate score for each (9-point scale). For NIH guidelines on scoring using a 9-point scale, please review the NIH guidelines:

"Scoring System and Procedure"
http://grants.nih.gov/grants/peer/guidelines_general/scoring_system_and_procedure.pdf

"Interpreting new application scores and critiques"
http://enhancing-peer-review.nih.gov/docs/scoring_and_critique_overview_June2009.pdf

1. **Significance.** The project addresses important problems or critical barriers to progress in cancer research or cancer disparities research. The aims of the project advance scientific knowledge, technical capability, and have near-term impact.

Significance Score: ____4_____

Strengths

The project addresses the breast cancer treatment-related problems among the particular population- Latina/Hispanic Women; provides a practical and hands-on approach through smartphone app; changes the target population from Latina BCS to more active patients and adjusts the app accordingly. All of the above could have a great impact on the breast cancer treatment among Latina/Hispanic Women. The proposed adjustments to the app could be a great improvement to the practical usage of the app.

Weaknesses

The current application seems well established, and the two adjustments seem minor compared to the current content. The adjustment on the target population is more significant, but besides the proposed new content to serve the purpose, the proposal did not provide more details about the adjustment to the current content to adapt to the change of target population.

2. **Innovation.** The application utilizes novel theoretical concepts, approaches or methodologies,

CONFIDENTIAL

instrumentation, or interventions.

Innovation Score: ____2_____

Strengths

The application provides a clear goal of improving the My Guide application by adjusting the target population to the active patients, adding new content of the diet and physical activity, as well as individually tailored content to each woman's phase of treatment and self-reported symptoms and concerns. The proposed trail study is also well organized to serve this goal.

Weaknesses

The application did not provide more details of the possible resources of the content, especially for the content of the individually tailored content. The example only provided the content designed for the categories that include treatment phase and symptoms, but not individual concerns.

3. **Investigators.** The PIs, collaborators, and other key personnel have the necessary experience and expertise to accomplish the goals of the proposed research project. The investigators have complementary and integrated expertise.

Investigators Score: ____3_____

Strengths

The team of investigators consists of bilingual interdisciplinary investigators with expertise in clinical oncology, breast cancer symptom management, patient education, behavioral interventions, and human computer interactions.

Weaknesses

The purpose of the experts in Phycology is not very clear. The proposed adjustment to the application did not include phycology content, especially how the patients' education or psychological help could be adjusted based on the adjustment of the target population.

4. **Approach** The overall strategy, methodology, and analyses is well-reasoned and appropriate to accomplish the specific aims of the project.

Approach Score: ____4_____

Strengths

The overall strategy, methodology, and analyses is very clear, well-reasoned and appropriate.

Weaknesses

The application proposed to use the same survey to study the outcome, and did not mention

CONFIDENTIAL

possible extra questions aimed to analyze the impact of the new content. The application also did not provide more details about the questions or data methods to compare the current proposed My Guide application.

5. **Career Development** There is a well thought-out and reasonable plan for career development of junior investigator(s). Mentor(s) have been identified and the appropriate mechanisms are in place to achieve career development goals outlined.

Career Development Score: ____3_____

Strengths

The proposed project will lead to several conference, manuscript, and grant submissions that will advance the careers of all investigators.

Weaknesses

The junior faculties do not have mentors or mentorship plan straightly from the senior PIs from the project.

6. **Community Engagement**. The partner organization(s) and their respective roles and contributions are clearly articulated.

Community Engagement Score: ___2______

Strengths

The project is a great collaborate of expertise from different fields. It is also a great opportunity to provide training to students. The application will be freely available to a large community and the project has a clear plan of the communities to be served as well as the preparation timeframe.

Weaknesses

-

7. **Training**. There will be significant opportunity for the training and research experiences for students and early stage investigators underrepresented in biomedical and cancer research

Training Score: __3_______

Strengths

The project provides detailed recruitment and training plan for students.

Weaknesses

The proposal did not provide direct mentorship or training plan from the project collaborate to the early stage investigators.

CONFIDENTIAL

8. **Potential to Generate Usable Data**. There is high likelihood that the proposed project or program will generate usable data, including preliminary data for future grant proposals or data for peer-reviewed publications
**Potential Data Score: ____7_____**<br><br>**Strengths**<br><br>**Weaknesses**<br>The data collected seems to be specially designed for the application.

9. **Potential for External Funding**. There is high likelihood that the proposed project or program will lead to more competitive grant applications to NIH and other types of foundation funding
**Potential Funding Score: ____5_____**<br><br>**Strengths**<br>The project has potential to be developed to an application with more contents and with larger-scale users.<br>**Weaknesses**<br>The My Guide application will be well established and available to publish at the end stage of the project.

ADDITIONAL REVIEW CONSIDERATIONS

As applicable for the project proposed, reviewers will address each of the following items, but will not give scores for these items and should not consider them in providing an overall impact/priority score.

Budget and Period of Support
Recommend as Requested<br>Recommended budget modifications or possible overlap identified:<br>•

ADDITIONAL COMMENTS TO APPLICANT

Reviewers may provide guidance to the applicant or recommend against resubmission without fundamental revision.

CONFIDENTIAL

Additional Comments to Applicant (Optional)
•

CONFIDENTIAL

ChicagoCHEC Incubator & Catalyst Grants Mechanism: Full Research Project

Application #: 01

Application Title: The My Guide e-Health Intervention for Latina/Hispanic Women Receiving Treatment for Breast Cancer (Short Title: My Guide)

Principal Investigator(s): Betina Yanez (NU), Frank Penado (NU), Alejandra Perez-Tamayo (UIC) Joanna Buscemi (UIC), Francisco Iacobelli (NEIU), Judy Guitelman (ALAS-Wings)

Cancer focus (select all that apply):

- ☐ Basic science
- ☐ Clinical
- ☐ Translational
- ☐ Prevention
- ☐ Control
- ☐ Behavioral
- ☐ Population research
- ☐ Policy

CONFIDENTIAL

OVERALL IMPACT

Reviewers will provide an overall impact score to reflect their assessment of the likelihood for the project to exert a sustained, powerful influence on the research field(s) involved, in consideration of the following nine scored review criteria, and additional review criteria. An application does not need to be strong in all categories to be judged likely to have major scientific impact.

Overall Impact *Write a paragraph summarizing the factors that informed your Overall Impact score.*
**Overall Impact Score: ____2_____**

SCORED REVIEW CRITERIA

Reviewers will consider each of the nine review criteria below in the determination of scientific and technical merit, as well as relevance to the partnership goals and objectives, and give a separate score for each (9-point scale). For NIH guidelines on scoring using a 9-point scale, please review the NIH guidelines:

"Scoring System and Procedure"
http://grants.nih.gov/grants/peer/guidelines_general/scoring_system_and_procedure.pdf

"Interpreting new application scores and critiques"
http://enhancing-peer-review.nih.gov/docs/scoring_and_critique_overview_June2009.pdf

1. **Significance.** The project addresses important problems or critical barriers to progress in cancer research or cancer disparities research. The aims of the project advance scientific knowledge, technical capability, and have near-term impact.

Significance Score: ___1______

Strengths

- Breast cancer accounts for 29% of all Latina cancer diagnoses and it is the leading cause of cancer-related death among Latina women. Latina women diagnosed with breast cancer are more likely to report poorer health-related quality of life (HRQoL), greater symptom burden, and greater cancer-related psychosocial needs than non-Latinas.

Weaknesses

- none

2. **Innovation.** The application utilizes novel theoretical concepts, approaches or methodologies, instrumentation, or interventions.

Innovation Score: _____2_____

Strengths

- The application is tailored to participants' reported symptoms and concerns. Treatments will be offered in Spanish which emphasizes culturally informed evidence-based strategies that are linguistically and culturally tailored to Latina women with breast cancer

Weaknesses

- none

3. **Investigators.** The PIs, collaborators, and other key personnel have the necessary experience and expertise to accomplish the goals of the proposed research project. The investigators have complementary and integrated expertise.

CONFIDENTIAL

Investigators Score: _____1____

Strengths Dr. Betina Yanez has expertise in cancer control, survivorship and health equity. Dr. Alejandra Perez-Tamayo is a breast cancer surgeon and Dr. Francisco Iacobelli is an expert in human-computer interactions. Dr. Joanna Buscemi is a licensed clinical psychologist with expertise in technology-supported behavioral interventions and obesity prevention and treatment. The team has the expertise to carryout the proposed aims

-

Weaknesses

- None

4. **Approach** The overall strategy, methodology, and analyses is well-reasoned and appropriate to accomplish the specific aims of the project.

Approach Score: ____2_____

Strengths

Aim 1. To develop a novel, adaptive and individualized version of the My Guide smartphone application for Latina women in active treatment for breast cancer.

Aim 2. To establish the feasibility of our novel, adaptive My Guide Smartphone application for Latina women receiving breast cancer treatment by implementing our study procedures (recruitment, assessment) and study conditions (My Guide vs. usual care control).

Weaknesses

- Limited Discussion on alternatives

5. **Career Development** There is a well thought-out and reasonable plan for career development of junior investigator(s). Mentor(s) have been identified and the appropriate mechanisms are in place to achieve career development goals outlined.

Career Development Score: ___1______

Strengths

- Well thought out and mentors are appropriate to guide the development of Dr. Buscemi

Weaknesses

- none

6. **Community Engagement**. The partner organization(s) and their respective roles and contributions are clearly articulated.

Community Engagement Score: _______1__

CONFIDENTIAL

Strengths

- CE is event throughout the application

Weaknesses

- none

7. **Training**. There will be significant opportunity for the training and research experiences for students and early stage investigators underrepresented in biomedical and cancer research

Training Score: __1 _______

Strengths

- In its first 3 years of this project, My Guide team will train 6 undergraduate interns, four graduate students, and one post-doctoral student.

Weaknesses

- None

8. **Potential to Generate Usable Data**. There is high likelihood that the proposed project or program will generate usable data, including preliminary data for future grant proposals or data for peer-reviewed publications

Potential Data Score: ____2_____

Strengths

- Data should generate publishable findings

Weaknesses

-

9. **Potential for External Funding**. There is high likelihood that the proposed project or program will lead to more competitive grant applications to NIH and other types of foundation funding

Potential Funding Score: _____3____

Strengths

-

Weaknesses

- If this pilot is successful, there is a potential for external funding, however the potential of this project is solely based on the data generated in the funding period.

CONFIDENTIAL

ADDITIONAL REVIEW CONSIDERATIONS

As applicable for the project proposed, reviewers will address each of the following items, but will not give scores for these items and should not consider them in providing an overall impact/priority score.

Budget and Period of Support
Click Here to Select<br>Recommended budget modifications or possible overlap identified:<br>•

ADDITIONAL COMMENTS TO APPLICANT

Reviewers may provide guidance to the applicant or recommend against resubmission without fundamental revision.

Additional Comments to Applicant (Optional)
•

CONFIDENTIAL

SCORES BY CRITERIA BY REVIEWER – CHICAGOCHEC FULL APPLICATIONS FUNDING MECHANISM										
Reviewer Name	Overall Impact Score	Significance	Innovation	Investigators	Approach	Career Development	Comm. Engagement	Training	Potential for useable data	Potential for External Funding
**APPLICATION 1 – THE MY GUIDE E-HEALTH INTERVENTION FOR LATINA/HISPANIC WOMEN RECEIVING TREATMENT FOR BREAST CANCER (MIGUIA)**										
Reviewer 1	2	1	1	1	2	1	2	2	1	1
Reviewer 2	1	1	1	1	1	1	1	1	1	1
Reviewer 3	3	4	2	3	4	3	2	3	7	5
Reviewer 4	2	1	2	1	2	1	1	1	2	3
**AVERAGE**	**2.00**	**1.8**	**1.5**	**1.5**	**2.3**	**1.5**	**1.5**	**1.8**	**2.8**	**2.5**

Note: some of the reviewers changed their scores during the in-person review after they submitted the written scores.
